# The Role of Tyramine β-Hydroxylase in Density Dependent Immunityof Oriental Armyworm (*Mythmina separata*) Larva

**DOI:** 10.3390/ijms20071553

**Published:** 2019-03-28

**Authors:** Hailong Kong, Chuanlei Dong, Wanghui Jing, Zhen Tian, Minyuan Zheng, Cheng Wang, Qiuli Hou, Yunxia Cheng, Lei Zhang, Xingfu Jiang, Lizhi Luo, Shude Zhu

**Affiliations:** 1 College of Horticulture and Plant Protection, Yangzhou University, Wenhui East Road, NO. 48, Yangzhou 225009, China; dong19940424@126.com (C.D.); jwhui123123@163.com (W.J.); 006783@yzu.edu.cn (Z.T.); zhengminyuan315@163.com (M.Z.); davionman@126.com (C.W.); houql@yzu.edu.cn (Q.H.); sdzhu@yzu.edu.cn (S.Z.); 2 State Key Laboratory for Biology of Plant Diseases and Insect Pests, Institute of Plant Protection, Chinese Academy of Agricultural Sciences, Yuanmingyuan West Road, No. 2, Beijing 100193, China; yxcheng@ippcaas.cn (Y.C.); leizhang@ippcaas.cn (L.Z.); lzluo@ippcaas.cn (L.L.)

**Keywords:** tyramine β-hydroxylase, *Mythimna separata*, octopamine, larval density, prophylactic immunity

## Abstract

High population density alters insect prophylactic immunity, with density-dependent prophylaxis (DDP) being reported in many polyphonic insects. However, the molecular mechanism for DDP remains unclear. In current study, the role of tyramine β-hydroxylase (Tβh) in the immune response of *M. separata* larvae that were subject to different rearing densities conditions was investigated. The tyramine β-hydroxylase activity of larvae from high density treatments (10 and 30 larvae per jar) was significantly higher than that of the larvae from low density treatments (one, two, and five larvae/jar). A *tyramine β-hydroxylase* (designated *MsTβh*) containing a 1779 bp open reading frame was identified. Multiple sequence alignment and phylogenetic analysis indicated that *MsTβh* was orthologous to the *Tβh* that was found in other lepidopterans. Elevated *MsTβh* expression was observed in larvae under high density (10 larvae per jar). Silencing *MsTβh* expression by the injection of dsRNA in larvae from the high density treatment produced a 25.1% reduction in octopamine levels, while at the same time, there was a significant decrease in phenoloxidase (PO) and lysozyme activity, total haemocyte counts, and survival against *Beauveria* infection 56.6%, 88.5%, 82.0%, and 55.8%, respectively, when compared to control larvae. Our findings provide the first insights into how *MsTβh* mediates the octopamine level, which in turn modulates the immune response of larvae under different population densities.

## 1. Introduction

Octopamine (OCT) is among a group of compounds that is known as biogenic amines. OCT is an invertebrate structural analog of vertebrate norepinephrine, and it is present in both vertebrate and invertebrate nervous systems [1]. OCT occurs as a trace amine in the nervous systems of most invertebrate species, including insects, where it plays a multifunctional role. OCT controls various peripheral functions, including modulation flight activity, visceral muscle, peripheral organs, and some sense organs [2,3]. In the central nervous system, OCT is vital in the regulation of motivation, maintenance of various rhythmic behaviors, social behaviors, and learning and memory [4]. OCT mediates starvation-induced hyperactivity in adult *Drosophila* [5]. OCT modulates muscle contraction and metabolism and fuel availability [6]. As a neurohormone, OCT modulates haemocytic nodulation in immune larvae and enhances phagocytosis [7]. OCT also increased the number of circulating hemocytes in *Spodoptera exigua* [8]. Our previous results also showed that OCT plays an important role in the larvae of *Mythimna sepatrata*, from high density conditions to be resistant to pathogens [9].

OCT is biosynthesized from tyrosine by two steps: tyrosine is decarboxylated by tyrosine decarboxylase to produce tyramine, followed by hydroxylation of tyramine by tyramine β-hydroxylase (Tβh) to form OCT [10]. The tyramine β-hydroxylase is the last and the rate-limiting enzyme in the biosynthetic pathway of OCT [2]. Davenport and Evans, in 1984 [11], postulated the hypothesis that the upregulation of Tβh activity that they observed might be due to an increase in *Tβh* expression. Lehman et al., 2000 [12] have demonstrated that elevated OCT levels during *Manduca sexta* development correlated with the induction of Tβh activity in the brain and ganglia during metamorphosis. Lehman et al., 2006 [13] have demonstrated that during honey bee behavioral development, an increase OCT levels in forager honey bee brain were correlated with the elevated expression of the gene encoding *Tβh*. Chatel et al., 2013 [14] found that stressful mechanical stimulation led to a significant increase in Tβh activity after 1 and 24h., although the level of OCT did not significantly increase in the same treatments. All these data exist seem to indicate that a link between Tβh enzyme activity or transcriptional activity, and OCT levels.

The oriental armyworm, *M. separata* (Lepidoptera: Noctuidae), is a major insect pest of grain crops in China and other Asian countries, and outbreaks have caused huge losses in annual crop production nationwide [15,16,17]. As an insect with typical phase polymorphisms, the variation of larval color polymorphism, survival rates, development rate, feeding behavior of larvae, and flight capacity, fecundity, and energetic reserves in adults from larvae reared at different larval densities have been found [18,19,20]. Meanwhile, Mitsui and Kunimi, 1988 [21] found that gregarious (high density) phases larvae were more resistant than the solitary (low density) phase ones when orally inoculated with nuclear polyhedrosis virus (PsNPV). Furthermore, our previous results also showed that resistance to entomopathogenic fungus *Beauveria bassiana* and bacteria *Bacillus thuringiensis* of larvae from high densities was higher than the larvae from low densities. Therefore, larvae that experience crowded conditions are more resistant to pathogens when compared to counterparts at low-densities, and this phenomenon is now known as “density-dependent prophylaxis (DDP)” [22]. The DDP affected the stability of the insect-parasite (or pathogen) interaction. Understanding the mechanism of DDP is of great importance in characterizing the population dynamics and controlling them by natural enemies [23].

Wang et al., 2016 [24] found that Tβh expression was related to aphid density, which affected wing polyphenism (winged or wingless), and aphids under high density conditions exhibited significantly higher *Tβh* transcription than those that were under low-density conditions. Our previous research suggested that immune indicators, such as phenoloxidase (PO) activity and total haemocyte counts and the level of OCT, increased in the larvae of *M. separata* from high-density conditions. Our previous research has demonstrated that exogenous OCT could significantly increase the PO activity and the number of total hemocytes of larvae [9]. Nonetheless, whether larval density can influence endogenous Tβh enzyme activity and transcriptional activity remains unclear, and whether the *Tβh* gene has a key role in insect density-dependent prophylaxis.

*M. separata* was investigated to gain further insight into OCT regulation, larval density-dependent prophylaxis of the armyworm. To accomplish this, we investigated the Tβh enzyme activity of larvae from different densities in rearing chambers. A full-length Tβh cDNA from *M. separata* was obtained and characterized, and mRNA expression was examined during the entire life cycle and in fifth-instar larvae that were reared under five different densities. Silencing the *Tβh* on the octopamine level and immune parameters were investigated. The study advances our knowledge of OCT-associated *Tβh* gene expression in the larval density dependent prophylaxis, and it provides information needed to explore the mechanism of octopamine on the larval immune function in *M. separata*.

## 2. Results

### 2.1. Tyramine β-Hydroxylase (Tβh) Activity

**Figure 1:**
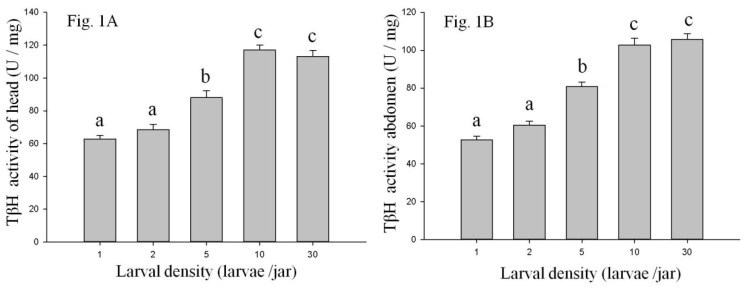
The activity of tyramineβ-hydroxylase enzyme of *M. separate* head (**A**) and abdomen (**B**) among larvae reared at one, two, five, 10, and 30 larvae per jar. Data are presented as the mean ± SE, *n* = 3. Different characters denote significant difference at *p* < 0.05.

Larval density had a significant effect on the Tβh activity in the head and abdomen (Figure 1A). As the larval density increased, the Tβh activity of the heads of larvae significantly increased. Tβh activity of larval heads from the 10 and 30 larvae/jar was highest, which was significantly higher than that of larval heads from one, two, and five larvae/jar. There was no significant difference in the Tβh activity of larval heads from one, two, and five larvae/jar. The same trend was observed in the Tβh activity of larval abdomens (Figure 1B). The Tβh activity of larvae from 10 and 30 larvae per jar was significantly higher than that of the larvae from one, two, and five larvae per jar.

### 2.2. Molecular Cloning of M. separata Tβh

**Figure 2:**
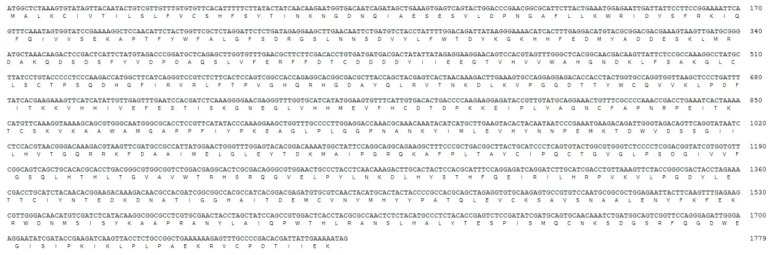
cDNA and deduced amino acid sequence of *M. separate Tβh*.

**Figure 3:**
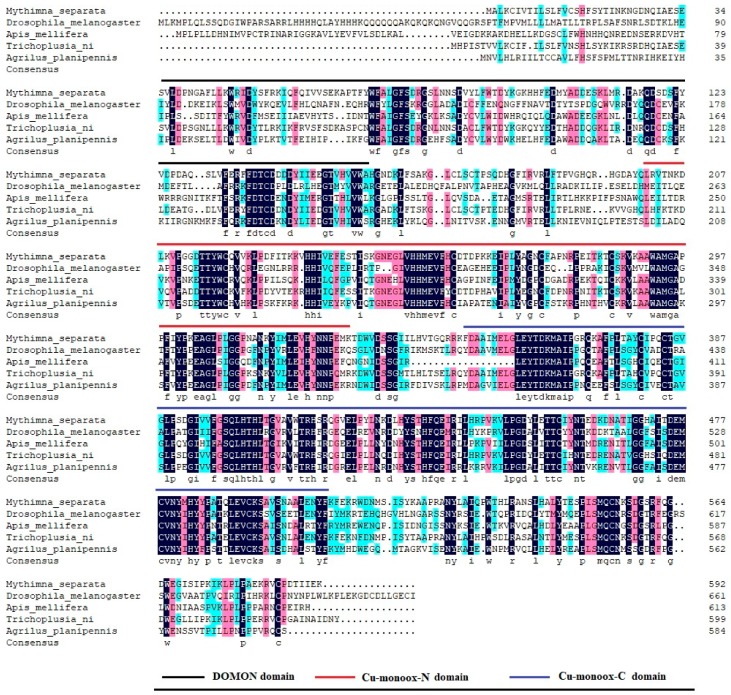
Multiple sequence alignment of *tyramine β-hydroxylase* (*MsTβh*) with other insect *tyramine β-hydroxylases*. The three different color lines represent the conserved domains, the black line is the DOMON domain, the red line the Cu-monox-N domain and the blue line Cu-monoox-C domain.

The cDNA sequence of *tyramine β-hydroxylase* of *M. separate* (*MsTβh*) is 1779 bp long and it contains a complete open reading frame (ORF) of 1779 bp that encodes a protein of 592 amino acids (Figure 2). A BLAST search and an alignment with other insect *tyramine β-hydroxylases* results showed the presence of three distinct domains: a DOMON domain (from position 37 to 157), a copper type II ascorbate-dependent monooxygenase N-terminal domain (Cu-monox-N, from positon 201 to 330), and a copper type II ascorbate-dependent monooxygenase C-terminal domain (Cu-monox-C, from positon 350 to 506) (Figure 3).

**Figure 4:**
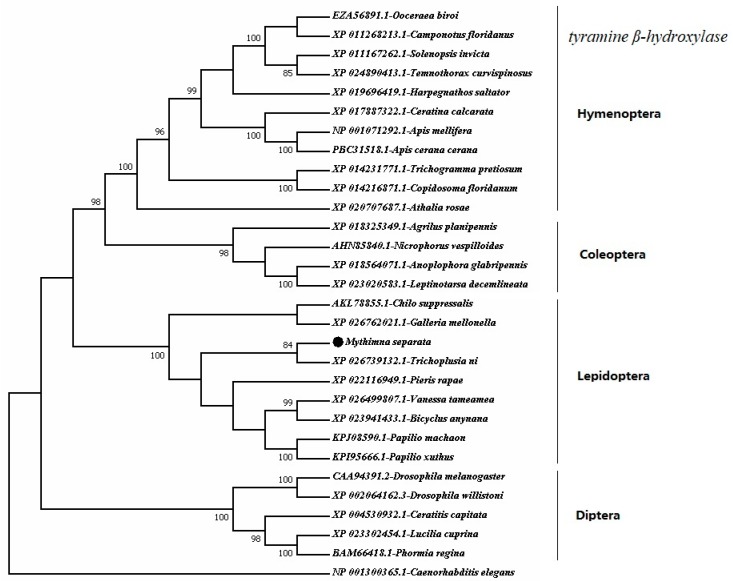
Phylogenetic tree of amino acid sequences of *tyramineβ-hydroxylases* from different insecta orders (Lepidoptera, Hymenoptera, Coleoptera and Diptera) was constructed by MEGA 7.0. *Caenorhabditis elegans tyramineβ-hydroxylase* was used as the out-group. The numbers at the nodes of the branches represent the level of bootstrap support for each branch.

A phylogenetic tree was constructed in MEGA 7.0 while using the amino acid sequences of *tyramine β-hydroxylases* from 30 insect species and *Caenorhabditis elegans tyramine β-hydroxylase* was used as the out-group (Figure 4). The tree showed that the insect *tyramine β-hydroxylases* are distributed in four main clades that correspond to Hymenoptera *tyramine β-hydroxylases* on one branch, Coleoptera on another branch, Lepidoptera on the third one, and Diptera on the fourth one. The *MsTβh* that was clustered with *TnTβh* (*Trichoplusia ni*, XP_026739132.1) and the 75% similarity was found in a BLASTP search of the National Center for Biotechnology Information databases.

### 2.3. Transcriptional Analysis of MsTβh from Larvae Reared under Different Densities

**Figure 5:**
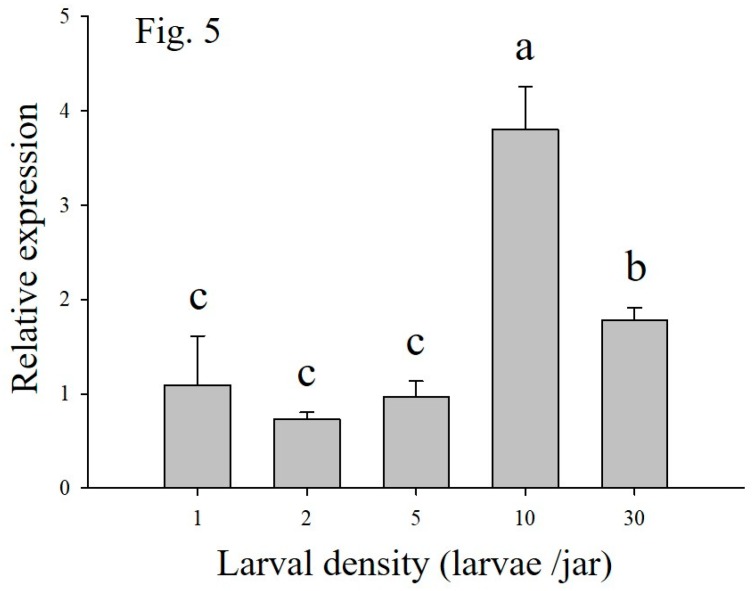
Transcriptional profiles of *tyramineβ-hydroxylase* (*Tβh*) of *M. separate* larvae reared at one, two, five, 10, and 30 larvae per jar. Data are presented as the mean ± SE, *n* = 3. Different characters denote significant difference at *p* < 0.05.

The transcriptional level of *MsTβh* was tested in the fifth instar larvae reared under one, two, five, 10, and 30 larvae per jar (Figure 5). The results showed that the highest transcription was found in the density of 10 larvae per jar, and it was significantly higher than those at higher (30 larvae per jar) or lower densities (one, two, five larvae per jar). However, there were not any significant differences among the densities of one, two, five larvae per jar.

### 2.4. RNAi Efficiency Analysis of MsTβh

**Figure 6:**
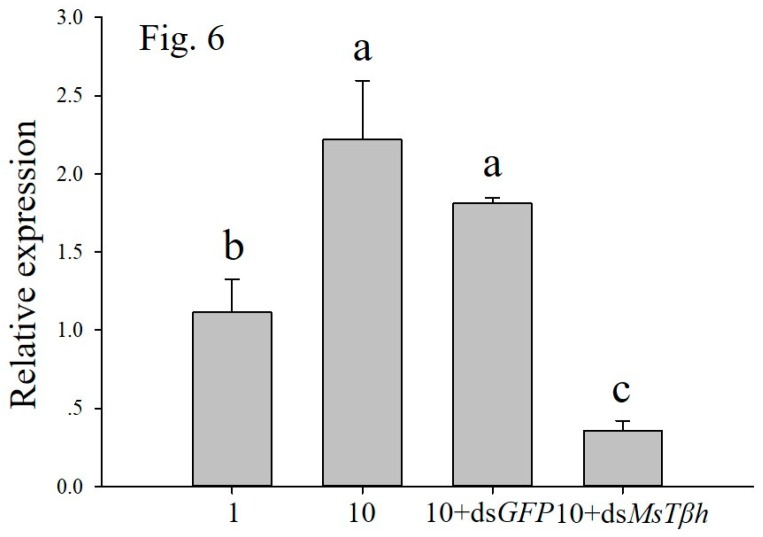
Effects of high density (10 larvae per jar) and 10 + ds*MsTβh* silencing on the *tyramine β-hydroxylase* (*Tβh*) transcript level of *M. separate* larvae. ds*GFP* has been as control. Data are presented as the mean ± SE, *n* = 3. Different characters denote significant difference at *p* < 0.05.

The high-density (10 larvae per jar) treated larvae led to a significant increase in the transcripts encoding *MsTβh* when compared to controls (1 larva per jar). The treatment of 10 (10 larvae per jar) + ds *MsTβh* led to reduced mRNAs encoding *MsTβh*, as compared to the high density enhanced transcript accumulation. None of the *MsTβh* transcripts were influenced by the 10 + dsGFP control treatments (Figure 6).

### 2.5. Effects of Injection dsMsTβh on OCT Level

**Figure 7:**
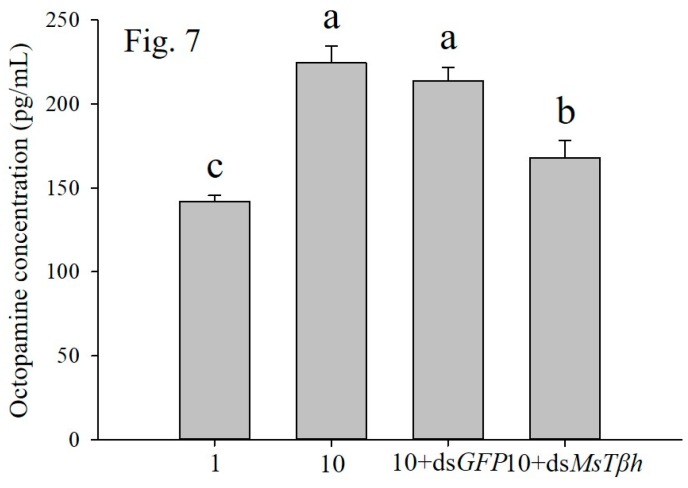
Effects of high density (10 larvae per jar) and 10 + ds*MSTβH* silencing on octopamine level of larvae. ds*GFP* has been as control. Data are presented as the mean ± SE, *n* = 3. Different characters denote significant difference at *p* < 0.05.

The OCT level of larvae from high-density (10 larvae per jar) was significantly higher than that of larvae from low-density (one larva per jar). The levels of OCT in larvae with 10+ ds *MsTβh* were significantly reduced by 74.9% when compared to the larvae from the density of 10 larvae per jar. OCT level was also not affected by the 10 + ds*GFP* control treatments (Figure 7).

### 2.6. Effects of Injection dsMsTβh on the Immunity

**Figure 8:**
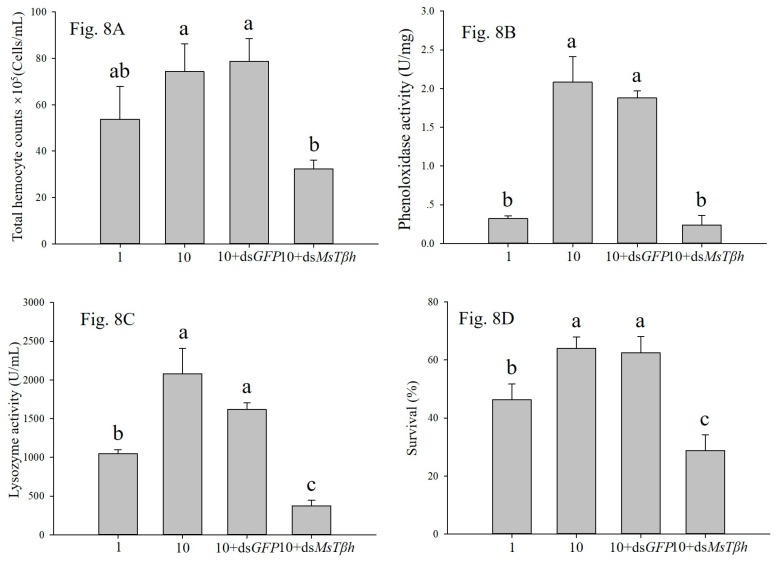
Effects of high density (10 larvae per jar) and 10 + ds*MsTβh* silencing on larval immune parameters and survival. ds*GFP* has been as control. (**A**) Total hemocyte counts; (**B**) Phenoloxidase activity; (**C**) Lysozyme activity; and, (**D**) Survival. Data are presented as the mean ± SE, *n* = 3. Different characters denote significant difference at *p* < 0.05.

The high density and ds*MsTβh* treatments influenced PO activity, lysozyme activity, total haemocyte counts, survival, and OA level of larvae. The high density led to increased PO activity, lysozyme activity, total haemocyte counts, and survival, which was reduced by about 56.57%, 88.46%, 82.00%, and 55.77%, respectively, in larvae that were treated with 10 + ds *MsTβh* (Figure 8). None of the PO activity, lysozyme activity, total haemocyte counts, and survival were affected by the 10 + ds*GFP* control treatments (Figure 8).

## 3. Discussion

OCT is biosynthesized from tyrosine by tyrosine decarboxylase and tyramineβ-hydroxylase enzymes. The tyramineβ-hydroxylase is a rate-limiting enzyme and it catalyzes the last step in the process of OCT biosynthesis. It plays a key role in the regulation of OCT synthesis. Some researches have demonstrated that increased Tβh activity was correlated with an increasing OCT level. During the *M. sexta* metamorphosis, it showed that elevated OCT level was correlated with the induction of Tβh activity in the brain and ganglia [12]. Mechanical stimulation stress led to an increase in Tβh activity and OCT level in *Periplaneta americana* [14]. Our results demonstrated that the Tβh activity of *M. separata* larvae from conditions of five, 10, and 30 larvae per jar were significantly higher than those of larvae at one and two larvae per jar. Kong et al. (2018) [9] found that, when compared with lower density larvae of *M. separata*, the larvae from high density conditions had significantly higher OCT levels. We inferred that high density led to an increase in Tβh activity and OCT level in *M. separata*. Our results also showed that the transcriptional levels of the *Tβh* gene in *M. separata* larvae from high density (10 and 30 larvae per jar) larvae were significantly higher than those of the lower density larvae (one, two, and five larvae per jar). This similar variation in the function of the density on the *Tβh* expression pattern was found in the Pea aphid *Acyrthosiphon pisum* [24]. Based on these results, we concluded that a link exists between Tβh enzyme activity or transcriptional activity and OCT levels in different densities of *M. separata* larvae.

With the transcriptome data of *M. separata*, a *Tβh* cDNA was amplified from the larvae. The cDNA of *MsTβh* contains an ORF of 1779 bp that encodes 593-amino acids. The alignment of multiple sequences between the *M. separata* and other Diptera, Coleoptera, Hymenoptera insects, and *Tβh* amino acid showed that *MsTβh* is highly conserved in these insects *Tβh*, including three domains: a DOMON domain, a copper type II ascorbate-dependent monooxygenase N-terminal domain (Cu-monox-N), and a copper type II ascorbate-dependent monooxygenase C-terminal domain (Cu-monox-C) (Figure 2). This indicates that *Tβh* is highly conserved across species.

In *Drosophila*, the knockout *Tβh* strain showed a decreased level of OCT [25]. In *Tribolium castaneum*, knockdown of *Tβh* led to reduced OCT level [26]. In the current study, we found that knocked down *Tβh* resulted in a decreasing OCT level in *M. separata*. Our results demonstrated that *Tβh* plays an essential role in the biosynthesis of OCT in *M. separata* larvae.

OCT is known to play critical roles in mediating insect immune response. Baines et al., 1992 [27] found that induced haemocyte phagocytosis and nodule formation in the cockroach *Periplaneta americana*. Kim and Kim, 2010 [8] reported that an injection of octopamine induced a significant increase in the total haemocyte count in the hemolymph. In response to bacterial infection, octopamine significantly enhanced both haemocytic phagocytosis and nodule formation in the *S. exigua* larvae [7]. Similarly, octopamine accelerated the removal of bacteria from haemolymph in the greater wax moth, *Galleria mellonella* [28]. In *M. separata* larvae, an injection of octopamine significantly stimulated the PO activity and total haemocyte counts [9]. We found that, in this study, that RNA interference-mediated silencing of the *MsTβh* exhibited markedly decreased PO activity, total haemoyte counts, lysozyme activity, and survival rate of the larvae. All of these results indicated that increasing OCT levels of larvae resulted in enhanced immunity. Octopamine was also involved in providing locusts with mobilizing fuel or lipid mobilization in flight [6]. In the high density conditions, improving immunity and flight capacity may cause the outbreak of *M. separata*.

Taken together, we demonstrated that Tβh is a key enzyme in the biosynthetic pathway of OCT, and that the larval densities of *M. separata* modulated its activity. After the silencing of *MsTβh* in high density larvae, the larval PO activity, total haemoyte counts, lysozyme activity, and survival were significantly decreased. Therefore, we concluded that *MsTβh* controls the biosynthesis of OCT, which in turn modulates the immunity of the *M. separata* larvae from different density conditions. However, further investigation is needed to understand how the octopaminergic signaling system modulates larval immunity, and significantly more research is needed in order to decipher the density dependent prophylaxis effect in insects.

## 4. Materials and Methods

### 4.1. Experimental Insects

The *M. separata* that were used in this study were a gift of Professor Jiang, Institute of Plant Protection, Chinese Academy of Agricultural Sciences.

A laboratory colony of *M. separata* larvae was reared on corn (*Zea mays* L.) leaves at 23 ± 1 °C, 70% relative humidity, under a 16 h light: 8 h darkphotoperiod. Adult *M. separata* were put in a 2-L capacity plastic cage that was equipped with a cotton pad soaked in 10% glucose solution (*w*/*v*) and allowed to oviposit on nylon gauze lining the plastic cage.

### 4.2. Larval Tyramineβ-hydroxylase Activity Assay at Different Densities

Enzyme was extracted in *M. separata* larvae that were reared at five different densities. After hatching, the larvae were reared at five density treatments of one, two, five, 10, and 30 larvae per 650-mL-capacity jar. The number of larvae for all density treatments was maintained at a constant level throughout the feeding period. Excess food was provided by adding fresh leaves every morning. All of the insects were maintained under the same conditions described above. The larvae bodies were collected as described above, when most of the larvae at a given density had developed to the second day of the fifth instar, and larvae were cooled to torpor on ice and then sterilized by swabbing them with 75% ethanol. The head and abdomen tissues from larvae were dissected under ice-chilled saline solution, frozen in liquid nitrogen, and stored at −80 °C. The tissues were thawed immediately prior to use, placed in a ground-glass homogenizer containing saline solution, and homogenized with 20 strokes by hand. The homogenate was centrifuged (6080 *g*, 30min) at 4 °C, and the resulting supernatant fraction was used as the crude enzyme source. The samples that were collected from groups of three larvae per density were pooled as one replicate. Three replicates were made at each density.

Tyramine β-hydroxylase activity was determined based on the Tyramine β-hydroxylase Elisa Kit (Nanjing Jiancheng Bioengineering Institute, Nanjing, China). A standard curve was made that was based on different concentrations of standard solutions. To the enzyme solution from the procedure described above (10 μL), sample diluents 40 μL and 100 μL HRP-Conjugate reagent were combined and incubated for 60 min. at 37 °C in microelisa wells. Each well was aspirated and then washed with a wash solution. Subsequently, 50 μL each chromogen solution A and B were added, incubated for 15 min. at 37 °C, and then 50 μL stop solution was added. The absorbency at 450 nm was measured while using a microplate reader (Power Wave XS2, Bio Tek Instrument Company of America, Ltd., Winooski, VT, USA).

### 4.3. Molecular Cloning of Tyramineβ-hydroxylase (Tβh) cDNA

Total RNA was extracted with Trizol Kit (Invitrogen, Carlsbad, CA, USA) from fifth-instar larvae of *M. separata* and the first-strand cDNA was synthesized using an oligo(dT)15 primer (Tiangen Biotech, Beijing, China) following the manufacturer’s instructions. The integrity of RNA was checked on an 1% agarose gel and Nanophotometer NP80 (IMPLEN, München, Germany) determined the concentration of RNA.

The complete open reading frames (ORFs) of *MsTβh* were amplified from larval cDNA. Primer (Tβh-F: 5′ATGGCTCTAAAGTGTATAGT 3′; Tβh-R: 5′ TTTTTCAATAATCGTGTCGG 3′) were designed based on the transcriptome data of *M. separata* with Primer Premier 6.0. The larval transcriptome dataset of *M. separata* has been released in the SRA database (SRP153130).

PCR amplification was carried out under the condition of one cycle at 95 °C for 3 min, 35 cycles at 95 °C for 30 s, 60 °C for 30 s, and 72 °C 45 s, followed by a final extension at 72 °C for 10 min. The purified PCR products were cloned into a pMD-18T vector (Takara, Dalian, China) and subsequently sequenced by Sangon Company (Shanghai, China).

### 4.4. Bioinformatic Analysis

Sequence alignments were carried out while using the BLAST software (http://www.ncbi.nlm.nih.gov). To investigate the phylogenetic relationship between *MsTβh* and *Tβhs* from other insect species, the amino acid sequences were aligned using the cluster algorithm ClustalW ver. 1.8, and a phylogenetic tree was constructed based on the alignment by MEGA 7.0 [29].

### 4.5. Transcriptional Profiles at Different Larval Densities

The transcriptional level of the *Tβh* gene was evaluated in *M. separata* larvae that were reared at different densities using the previous methods as described above. All of the samples were snap frozen in liquid nitrogen before being stored at −80 °C for later use. The total RNA of larvae from different densities was extracted using a Trizol Kit (Invitrogen, Carlsbad, CA, USA). First-strand cDNA was synthesized using an oligo (dT) 15 primer (Tiangen Biotech) and 1μg of total RNA as the template in a final volume of 10 μL. cDNA that was prepared from total RNA was used as the template for real-time quantitative PCR (qPCR) with an ABI PRISM 7500 Real-Time PCR System (Applied Biosystems Foster, CA, USA). *MsTβh* primers (F: CCAAGACCATGGCTTCATCAG; R: ACCAGTAGGTAGTGTCTCCT) were designed; β actin was selected as the reference gene (F: GCGACATCAAGGAGAAGCTC; and, R: TTCCGATGG TG AT GACTTGA).

Three biological replications were used for qPCR analysis while using a 20-µL total reaction system containing 0.1 µg total RNA, 0.8 µl primer mix containing 10 µM of each forward and reverse gene-specific primers, 0.4 µL ROX Reference Dye II (50×), 2 µL cDNA, 10 µl SYBR Premix EX TaqTM II, and 6 µL H_2_O, following the One Step SYBR Premix Ex TaqTM II Kit instructions (Takara Biotechnology Dalian Co., Ltd., Dalian, China). The thermal cycling conditions were 15 min. at 95 °C, followed by 40 cycles of denaturation at 95 °C for 30 s, annealing at 58 °C for 30 s, and elongation at 72 °C for 35 s. Relative quantifications were calculated using the 2^−ΔΔ*C*t^ method [30].

### 4.6. RNA Interference of Tβh

Gene-specific primers tailed with T7 promotors were used to amplify the target region for the synthesis of double strand RNA (dsRNA) of *Tβh*. The amplified products were used as the templates in the synthesis of ds *MsTβh* using the Transcript Aid T7 High Yield Transcrition Kit (Thermo Fisher Scientific, Waltham, MA, USA) based on the manufacturer’s instructions. The 1% agarose gel electrophoresis tested the integrity of dsRNA, and a Nanophotometer NP80 measured the concentration (IMPLEN). The dsRNA was diluted with DEPC-treated water to four concentrations, 4000, 2000, 1000, and 500 ng/µL, from which 2000 ng/µL was selected as the final concentration, according to the mortality and efficiency of gene silencing data.

On the second day of the fourth larval instar, larvae from the density of 1 and 10 larvae per jar were used for the RNAi experiment. Three microgramsof *dsMsTβh* or *dsGFP* were injected into the proleg of larval third abdominal segment while using a microinjector. Four treatments were set as following: (1) one larva per jar (control); (2) ten larvae per jar; and, (3) ten larvae per jar + ds*GFP* (4) ten larvae per jar + ds*Tβh*. To confirm the dsRNA expressed, the differently treated larvae were used for the RNAi assessment by qRT-PCR after 72 h. The method of qRT-PCR was performed, as described above. For the RNAi, ds*GFP*-injected larvae served as control. Table 1 lists all of the primers used.

On the 72 h after dsRNA injection, the three aspects of immune function were assayed for treatment larvae. To perform the assay, the larvae were cooled to torpor on ice and then sterilized by swabbing with 75% ethanol. An abdominal proleg was cut with a fine scalpel, and haemolymph was collected with microcapillary pipette and then transferred into a 1.5-mL centrifuge tube on ice to prevent melanization. Haemolymph samples were collected from the injected larvae, and haemolymph from the same treated larvae were pooled as one biological replicate for the assays to determine PO and lysozyme activities and for haemocyte counts. Three biological replicates were established for every treatment. All of the samples were then frozen at −80 °C until evaluation for the immune function.

PO activity was assayed as described by Kong et al., (2018) [9]. 50 µL haemolymph was added to 150 µL phosphate buffer (pH = 7) in a plastic tube. PO activity was assayed spectrophotometrically by adding 1500 µL 0.1M L-dopa and 1000 µL phosphate buffer (pH = 7) to 200 µL of haemolymph phosphate solution. Changes in the absorbance of the mixture within 3 min. at 492nm were measured while using a time-dependent program and a lambda 25UV/VIS spectrometer (Perkin Elmer Company, USA). The protein content was measured in the samples according to the method that was described by Bradford et al. (1976) [31]. PO activity is expressed as PO units per milligram of protein, where 1U was the amount of enzyme that was required to increase the absorbance by 0.001min^−1^. The total haemocyte count was determined by adding 5 µL haemolymph that had been stained with Giemsa for 5 min. to the counting chambers of a haemocytometer. The haemocytes in the central square and four corners were counted under phase-contrast illumination and then averaged to give an estimate of the number of haemocytes. Lysozyme activity was determined with a lysozyme kit (Nanjing Jiancheng Bioengineering Institute, Nanjing, China) based on the user’s instructions. First, 0.2mL haemolymph was mixed in a test tube with 2mL bacterial culture medium, and the two absorbance values at 530nm were determined in a UV-2000 spectrophotometer (Unico Instrument Company of Shanghai, Shanghai, China) after 20 and 140 s, respectively. The lysozyme activity was determined by the two values, according to the user’s instructions.

For resistance bioassays after RNAi, four groups of 180 different treatment larvae were exposed to the LC_50_ dose of *B. bassiana* (3.6 × 10^7^ spore per mL) by collectively dipping them in the spore solution for 5s, followed by air-drying at room temperature. The survival rate was recorded daily until all larvae either died or pupated.

### 4.7. Data Analysis

Data for different density treatments to test larval enzyme activity, gene expression, and RNA interference were analyzed using one-way analysis of variance (ANOVA), with the Duncan’s multiple range test for multiple testing. All of the statistical analyses were performed with SPSS 10.0.

## Figures and Tables

**Table 1:** Sequence of primers used for double strand RNA (dsRNA) synthesis and quantitative PCR (qPCR) of RNAi in *M. separata tyramineβ-hydroxylase*.

Primer Name	PRIMER SEQUENCE	Length
dsMsTβH-F	TCGATGCCGCCATTATGGAA	354bp
dsMsTβH-R	TCGTGGCGTTGTCTTTGTCT	
dsGFP-F	AAGGGCGAGGAGCTGTTCACCG	657bp
dsGFP-R	CAGCAGGACCATGTGATCGCGC	
qMsTβH-F	CCAAGACCATGGCTTCATCAG	128bp
qMsTβH-R	ACCAGTAGGTCGTGTCTCCT	
βactin-F	GCGACATCAAGGAGAAGCTC	126bp
βactin-R	TTCCGATGG TGATGACTTGA	
